# Facilitators and barriers of wet nursing: a qualitative study with implications for emergencies

**DOI:** 10.3389/fnut.2025.1456675

**Published:** 2025-05-09

**Authors:** Khadija Abdelrahmman, Bindi Borg, Karleen Gribble, Seema Mihrshahi

**Affiliations:** ^1^Master of Public Health (MPH), Department of Health Sciences, Faculty of Medicine, Health and Human Sciences, Macquarie University, Sydney, NSW, Australia; ^2^Department of Health Sciences, Faculty of Medicine, Health and Human Sciences, Macquarie University, Sydney, NSW, Australia; ^3^School of Nursing and Midwifery, Western Sydney University, Sydney, NSW, Australia

**Keywords:** wet nursing, breastfeeding, cross nursing, emergencies, infant and young children feeding in emergencies

## Abstract

**Introduction:**

Supporting recommended breastfeeding practices, including wet nursing in emergencies, is crucial to providing safe and nutritious food for infants and young children to support their health and wellbeing in critical resourced situations. However, it is only now that practical guidelines for implementing wet nursing in emergencies are being developed (by UNICEF). As there is very little literature on wet nursing in contemporary settings, this study aimed to explore current wet nursing experiences to identify the facilitators and barriers of the practice, especially in emergencies.

**Methods:**

Online semi-structured interviews were conducted from September to November 2023 with eight breastfeeding counselors and advocates. Transcripts were inductively analyzed using a reflexive thematic approach.

**Results:**

Seven themes were identified that reflect the factors affecting wet nursing. These included “wet nursing saved lives,” “breastfeeding is valued,” “infant formula is normalized,” “risk of infection transmission,” “all stakeholders' acceptance,” “counseling approach,” and “planning for implementation.” These results suggest that facilitators and barriers that generally affect maternal breastfeeding in emergencies also influence wet nursing. Certain obstacles may have a particular impact on wet nursing, including the risk of infection transmission, negative attitudes toward wet nursing and the need for culturally sensitive counseling to mediate wet nursing.

**Conclusion:**

Promoting wet nursing is an Infant and Young Children Feeding in Emergencies recommendation (IYCF-E) for non-breastfed infants. Implementing wet nursing programs can be in line with supporting good breastfeeding practices in emergencies. The results of this study can guide practical steps for implementing wet nursing and future investigation in different contexts.

## Introduction

Wet nursing refers to the practice of children being breastfed by women other than their biological mothers in a variety of circumstances, particularly when mothers are unable to breastfeed due to illness or death (1, 2). Wet nursing was a common practice and well-recognized profession in many cultures from antiquity until the beginning of the 20th century, when both the demand and supply of wet nurses declined (3). However, the practice persists, particularly among close relatives and friends in some communities (4). It is worth noting that wet nursing can include different practices such as cross-nursing. This is where the child is still breastfed by the biological mother, but other women also routinely breastfeed the child whenever the biological mother is temporarily unavailable due to work or study, so (5).

Interrupted breastfeeding due to mother-child separation, particularly during humanitarian emergencies, imposes risks of infection, malnutrition and even death on children, especially those aged under 6 months (6, 7). Introducing infant formula has inherent risks for infants in this age group in both low- and middle-income countries (LMIC) and high-income countries (HIC) (8–10). Formula-fed infants are at greater risk in the short term, especially for gastrointestinal and respiratory tract infections, as well as longer-term risks to their health and development, including non-communicable conditions such as obesity and diabetes mellitus (9, 10). These risks are exacerbated in emergencies where infrastructure is interrupted, resulting in a lack of clean water and facilities for washing and sterilizing equipment needed for infant formula preparation (11, 12). Moreover, water-borne diarrheal outbreaks are common during emergencies and children under 5 years are the most vulnerable to these infections (12). A case-control study during flooding in Botswana 2005/2006 found that the probability that case children (0–5 years) had been breastfed in the seven days before getting diarrhea was thirty times lower than that of control children (13). After the Yogyakarta Earthquake in 2006, infants whose households received infant formula donations had about double the risk of diarrhea in comparison to those who did not receive formula (11).

Accordingly, the Operational Guidance on Infant and Young Children Feeding in Emergencies (OG-IFE) states that wet nursing should be considered a first choice when the biological mother cannot breastfeed or provide expressed milk to her infant (6). However, to date, there have been no guidelines for wet nursing implementation in emergencies (14). UNICEF is currently in the process of addressing that gap. The goal of the forthcoming “Wet Nursing Guidance on Infant and Young Child Feeding in Emergencies (IYCF-E)” (Wet Nursing Guidance) is to ensure the safety, health, and growth of infants and young children by assisting in the facilitation of wet nursing as a safe emergency response measure.

Previous literature on infant and young children feeding in emergencies discussed general factors related to following the OG-IFE and focused mainly on supporting maternal breastfeeding and challenges such as violating the International Code of Marketing of Breast-milk Substitutes (11, 15–19). On the other hand, literature specifically discussing wet nursing consists mostly of historical reviews that investigated the Western experiences of wet nursing as a profession (20). Many of those papers reflected the negative experiences of discrimination and prejudice against wet nurses, yet some articles highlighted the positive role of wet nurses in several cases (20–23). Contemporary literature on wet nursing explored attitudes to the practice in African and Muslim communities where wet nursing may still be common among close relatives (20, 24–26). Azad et al. (1) and Burrell et al. (14) described the wet nursing program supported by Save the Children in the Rohingya refugee camps in Bangladesh during 2017. These authors discussed challenges including misunderstandings between wet nurses and the nursed children's families and logistic barriers such as night feedings (1, 14). Nonetheless, research on wet nursing in emergencies remains scant. This present study aims to explore contemporary experiences of wet nursing in different cultures, particularly during emergencies, and to identify facilitators and barriers of the practice. This knowledge will assist in understanding the factors that should be considered in implementing the practice of wet nursing as a first alternative to maternal breastfeeding to provide safe and nutritious feeding for children if their mothers cannot breastfeed them during emergencies.

## Methods

### Recruitment

Participants were recruited using purposive sampling. Potential participants were wet nurses or others with experience of wet nursing (for example, as a mediator of the practice), especially in emergencies, who could communicate in English. We were interested in exploring direct wet nursing and so, did not include situations where a child was fed another mother's expressed breast milk. The definition of emergency in this study is a general urgent situation where the biological mother is unable to breastfeed her child due to her illness and includes humanitarian situations following natural disasters or armed conflicts. Potential participants were identified and contacted through the Technical Advisory Group (TAG) convened to assist UNICEF in developing their Wet Nursing Guidance. TAG members identified participants from breastfeeding advocates and nutritionists who previously worked in non-governmental organizations supporting breastfeeding mothers in emergencies. The study protocol was approved by the Macquarie University Human Research Ethics Committee (Reference No: 520231433852217).

### Data collection

Semi-structured interviews were conducted by the first author (KA) online via Microsoft Teams between September and November 2023. After obtaining verbal consent, participants were asked about the circumstances in which they practiced or mediated wet nursing, followed by questions about the factors that affected the practice and how to mitigate the obstacles encountered in their experiences. The semi-structured interview had a simple guide of four questions allowing the interviewer to further probe participants as required and its time ranged from 20 min to an hour. The interview guide is included in Supplementary File 1.

With the participants' consent, the interviews were recorded via the online platform Microsoft Teams. Transcripts were automatically generated by Microsoft Teams and then anonymised with random numeric codes after the interview. Transcripts were then double-revised against the recordings to correct misinterpreted words. Descriptions of participants' responses, like pauses and laughing, were also included to increase the quality of the transcripts for accurate interpretation of the meaning (27). The anonymised transcripts were imported to NVivo (Release 1.7.1) for analysis.

### Analysis

Transcripts were inductively analyzed by KA using a reflexive thematic approach guided by the work of Braun and Clarke (28). After data familiarization, the transcripts were coded, generating over 150 code labels. The lead researcher adopted an inductive approach where the data itself drives coding so that the analysis would reflect the participants' experiences with wet nursing. Codes were clustered according to similar and reoccurring meanings to develop broad themes and subthemes and were rearranged, defining and naming themes. The resulting themes and subthemes were organized with definitions, discussed with the whole authorship team, and agreed on the final version.

## Results

Fourteen participants were contacted through the TAG and eight women were successfully interviewed. No new themes were identified after the seventh participant, suggesting that data saturation may have been reached. All study participants had at least a bachelor's degree. They were specialized nutritionists, lactation counselors, or breastfeeding advocates volunteering in non-governmental organizations. Most participants acted as wet nurses in general or humanitarian emergencies. They drew their experiences from ten different countries (some participants mediated wet nursing in several countries). Participants' characteristics are summarized in Table 1.

**Table 1:** Characteristics of the study participants.

**Characteristic**	**Specification**	**Number of participants**
Age range	30–40 40–50 50–60 60–70	2 3 2 1
Education level	Bachelor Master's PhD	2 4 2
Experience with wet nursing	Mediated wet nursing in: Humanitarian emergency	6
	General emergency	2
	Acted as wet nurse	5
Regions	Southeast Asia Pacific Middle East Africa Europe	

**Figure 1:**
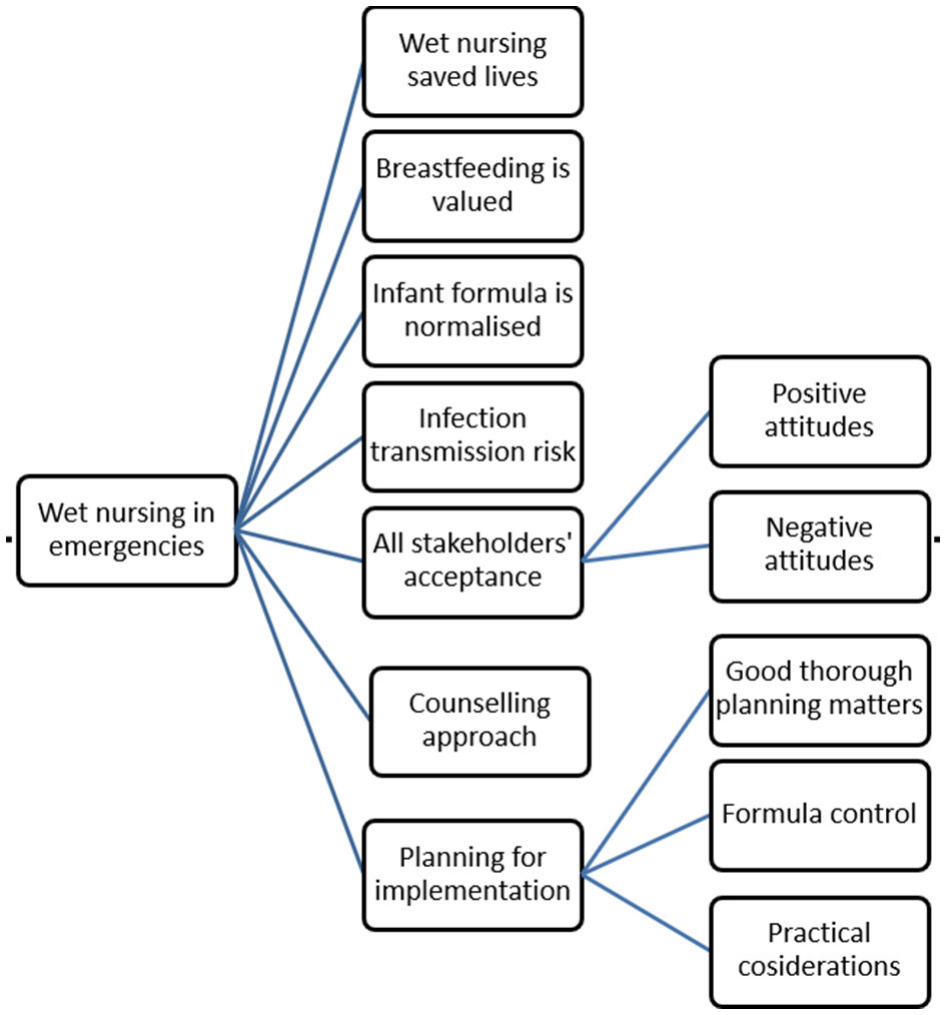
Concept map of themes and subthemes.

Seven themes were identified in the data as shown in Figure 1. These themes were: wet nursing saved lives, breastfeeding is valued, infant formula is normalized, risk of infection transmission, all stakeholders' acceptance, counseling approach, and planning for implementation. There were some sub-themes to all stakeholders' acceptance and planning for implementation themes.

### Wet nursing saved lives

Participants who practiced or mediated wet nursing in emergencies described the profound effect that wet nursing could have on infants who were malnourished and in precarious situations, such as in refugee camps with minimal resources. One participant described the pediatrician's reaction when seeing how the orphaned infant's health had improved: “*the pediatrician told me, even after like one day, he actually noticed that there [was] a difference in the color of the baby's skin, how she became […] responsive […] he told me, like, “you saved that baby” […] and she didn't [need to] go to the hospital.”* Participant 43 (this is a numerical code that was added for differentiation between participants' quotes and not related to the order of their interviews or the codes used for the transcripts to maintain high level confidentiality).

Another participant found wet nursing remarkably beneficial for underweight infants: “*The infants got better. And even the small one, she survived, she gained really, really quickly weight back […] I mean, it's serious for survival in certain very difficult situations. It's immensely useful for the health of the child. I mean from a very, very difficult background.”* Participant 31

### Breastfeeding is valued

Almost all participants believed that a major facilitator to wet nursing is when breastfeeding is already highly valued and where the importance of breastfeeding is appreciated, especially in emergencies: “*[To implement wet nursing] you have to really search for someone who is a breastfeeding advocate, who is willing to deal with the idea and implement the idea on the ground.”* Participant 18

This pro-breastfeeding attitude could be from mothers, caregivers, potential wet nurses or staff working with breastfeeding women, including counselors, midwives, nurses or general practitioners. For example, one participant passionately explained why she wet nursed her baby relative: “*I am myself, a wet nurse mother. […] I love wet nursing. I advocate for wet nursing.”* Participant 18

Valuing breastfeeding was based on sound knowledge of breastfeeding in general and understanding the risks of infant formula. A participant explained how this knowledge facilitated wet nursing practice: “*I think having an awareness that infant formula is not a sterile product and it's difficult to prepare well safely and it introduces risk of an increased susceptibility to infectious diseases.”* Participant 51

In a couple of situations, participants stated how mothers who were exclusively breastfeeding refused to use infant formula and sought wet nursing when they were not able to continue breastfeeding their own children.

Understanding breastfeeding's value not only facilitated wet nursing but also could motivate and assist caregivers and wet nurses to overcome situations and requirements that would otherwise be a barrier: “*[to accept screening], the [wet nurse] mother has to be a lover to breastfeeding.”* Participant 18

Knowledge about breastfeeding's importance and infant formula risks was viewed as important to share as a part of counseling related to wet nursing: “*When women have trusted health professionals, explain to them that formula feeding will put their baby in danger, then I think they are much more willing… I mean, the barrier disappears*.” Participant 51

To illustrate how to target this knowledge, a participant described an experienced counselor as the one who can respond to all caregivers' concerns. “*Before [introducing wet nursing], you really have to do counseling through the family because of the misinterpretations […] That's part of the counseling.”* Participant 97

### Infant formula is normalized

Preferring infant formula was expressed as a common barrier to wet nursing because of the perceived convenience associated with formula feeding: “*Many women, because they wanted to go back to work or they had lots of other responsibilities, it was very mainstream to provide the formula with the bottle.”* Participant 31

Almost all participants thought this trend was due to the extensive infant formula marketing that has reached almost everywhere, including rural communities: “*In [my culture], we have formula preferences. Unfortunately, the industry has infiltrated our culture. Wet nursing, I consider, is a part of [our] culture, but unfortunately, right now it shrank because number one, in my opinion, is families right now value formula more than breast milk.”* Participant 18

“*They have their concerns because they live in the mountains. So now, there the marketing of formula milk is being introduced to them. So, that's the sad part, but that's the reality.”* Participant 10

Participants thought that this marketing effect influences not only the public but also health professionals who may recommend infant formula in an attempt to avoid any risk of infection transmission that may be associated with wet nursing: “*Sometimes there are concerns from the health workers about wet nursing [and] how we make sure the health of the wet nurse mother, because sometimes maybe she has an illness that we don't know. Are we screening them properly? It's something that is scary for some other people to think. ‘OK, then rather risking that the baby got infected, then just keep formula.”'* Participant 63

### Risk of infection transmission

Related to the above-described health professionals' concerns, almost all participants found that the possible risk of infection can be the main barrier to wet nursing, especially when an infection is prevalent in a specific area. The participants named two infections: human immunodeficiency virus (HIV) and Human T-lymphotropic virus-1 (HTLV-1).

### Fear of infection transmission

Participants reported that concerns about infection transmission were expressed by both caregivers and health professionals, with the misconception that any infection can be transmitted from the wet nurse to the nursed child. Participants thought that this misconception started after the emergence of HIV during the 1980s and that health professionals pay attention to these concerns as part of their duty of care. Talking about the fear of infection transmission, one participant said: “*Infectious disease transmission was something everyone mentioned.”* Participant 80

“*In the 90s, late 90s, early 2000s, everyone was terrified of breast milk […] like, Oh my God, it could be dirty and contaminated and dangerous.”* Participant 51

Yet, participants, as breastfeeding counselors and advocates, described these concerns as exaggerated because the risk of blood-borne infection transmission via breastfeeding is low and can be managed.

### Controlling the risk of infection

HIV was mentioned as the primary pathogen of concern. HIV testing is one way of mitigating the risk of HIV transmission; however, participants mentioned two problems with testing in emergencies. First, testing is not available during emergencies, especially if infrastructure is heavily damaged or in remote areas: “*In the situation where it was a rural village, there wasn't access to testing. […] We had traveled by boat down the river for four hours, and then we had walked sometimes through like hip-deep mud for another two or three hours to get into these villages. They were very remote.”* Participant 51

Second, HIV testing can be intrusive or arouse discrimination against disadvantaged people: “*We couldn't test because there were a lot of stigmas associated with the refugees that were coming. They were already unwelcome, and it was not allowed … to test them because had they been positive for HIV if we knew the prevalence in the camps, those camps would have been further discriminated against; it already was an issue.”* Participant 31

The approach in these cases was to confidentially collect history from potential wet nurses to assess the risk they had HIV. It was noted that this should be carried out by a culturally sensitive counselor with confidentiality assured: “*We had a wet nurse who came forward, and she told us she was willing to support us… because we couldn't test her for HIV, I asked one of my woman's staff to ask her some questions about her life that could potentially determine whether she was at a high risk of having contracted HIV and also, we asked her about her husband […] we explained to the woman that those answers would only remain with us and had there been any issue, would not have said to anybody.”* Participant 31

In some contexts, participants noted that pregnant women usually have antenatal care with screening for many infections, including HIV. Where this has occurred, potential wet nurses would know their HIV status and infection risk via wet nursing is diminished.

On the other hand, participants who worked in extreme humanitarian conditions saw that even in high HIV prevalence areas, and even where HIV testing is not available, providing breast milk via wet nursing may still be the least risky option if high morbidity and mortality risk of other diseases like diarrhea and acute respiratory tract infections are present: “*…[it] doesn't mean that HIV wasn't highly prevalent, but if that baby at that age had not been fed breast milk, the likelihood of it surviving would have been quite low. And so, where testing is not available, you need to make a risk-benefit analysis.”* Participant 51

### All stakeholders' acceptance

Caregivers, wet nurses, their families and health professionals' attitudes toward wet nursing were found to be crucial elements in the practice. Factors enhancing positive and negative attitudes are described in the next section.

### Positive attitudes

Participants were of the view that wet nursing would be more accepted where the practice is still common as part of traditional practices, for example, in some Indigenous and rural communities: “*In the [Indigenous] community, in their tribe, when the woman has to work, they have a neighbor [who] wet nurses, which is normal for them.”* Participant 10

It was noted that Muslims are familiar with wet nursing as the Prophet Mohammed was wet nursed for two years by Halima Al-Sadia: “*They told me that since the community was Muslim as they had in their oral teachings … that the Prophet [Mohammed] was wet nursed.”* Participant 31

Interestingly, wet nursing was accepted in communities where the practice was uncommon as a spontaneous response to the needs of orphaned infants. While being uncommon in her culture, one participant explained that she wet nursed an orphaned refugee infant because of her care as a mother: “*It wasn't planned […] They put like this artificial nipple from the bottle inside of [the infant's] mouth. And she was not reacting to the flow of the milk. And then just as a mother, I think, and as a woman, I just ask, like, if it is OK with the aunt that I hold the baby … And since the baby does not eat. It's not responding very well if it's ok for them that I offer my breast instead.”* Participant 43

Other facilitators of wet nursing acceptability were suggested, such as viewing wet nursing as more feasible than formula feeding, which requires equipment for reconstitution, washing, sterilization and preparation. The availability of lactating relatives or close friends was a common factor in several described wet nursing and cross-feeding experiences: “*My best friend [had a health problem]. She couldn't breastfeed anymore because she was under medication, and I think the medication wasn't compatible with breastfeeding… So, I just offered that I could wet nurse her baby, and she would just visit me, and I wet nursed her baby.”* Participant 18

Community cohesion and the desire to help others enhanced the acceptability of wet nursing in two ways. First, mothers wanted to help other mothers or orphaned children, and this was a strong motive even when support (like additional food) was provided to wet nurses. Second, this cohesive community spirit was seen when help in caring for her children was offered to wet nurses so they could more easily breastfeed the orphaned infants: “*I don't know if some of them accepted … because they were getting some food, but… I felt, the ones we talked to, they were happy to do it. I felt some excitement about doing this, about caring for the child, so it wasn't only about the money or the food, which is valued as money.”* Participant 31

It was noticed that mothers with children older than 6 months were more willing to act as wet nurses, while women who had previous successful breastfeeding experiences appeared more open to wet nursing either as a wet nurse or allowing their infant to be wet nursed. Interestingly, one participant referred to the mutual kindness that both donor and recipient mothers would have*: “The recipient mother said she would be honored to have her child fed by this other mother. So, there was this tremendous respect for the gift of giving milk and also receiving another mother's care. And I think often the generosity seems to be always framed as going one way when a mother who's a recipient mother also has to open her heart to the receipt of the milk.”* Participant 80

Finally, one participant suggested that the presence of milk banks may help communities recognize wet nursing as a valid option for infant feeding: “*A few years ago, we supported … establishment of the first bank for human milk…. This bank is serving mostly children who are in intensive care units… Through that opening, we also did a lot of, you know, public awareness raising campaigns among women, especially those who are having children, who are breastfeeding to become donors, right. So, I would also say that now, this idea ‘giving your milk for somebody else, including for wet nursing', [became] more acceptable among the general population than it was perhaps like seven years ago, but now even among… hospitals, because of our work, some practices are [now] more acceptable.”* Participant 43

### Negative attitudes

Factors described as negatively affecting wet nursing differed according to context. For example, common Western views, including breastfeeding as an intimate act and breast milk as a body fluid, were noted as factors that could add to the fear of contamination and infection transmission. On the other hand, in Muslim cultures, milk kinship was sometimes a barrier to wet nursing because of concerns about unintended marriage between the nursed children and their milk siblings and the idea that wet nursing would reduce marriage chances. It is worth noting that while milk kinship is a universal Islamic rule that was mentioned in the Holy Quraan, there are differences in the religious interpretation between Islamic doctrines of the number and method of feedings (direct wet nursing or expressed milk) which necessitates the prohibition of marriage: “*I think people were much more open to the fact that the interpretation of religious rules about cross-feeding and sharing milk was very varied. There was not one interpretation of what was Haram or whatever, and they understood there were very big regional and cultural differences in [the] religious interpretation of those religious rules.”* Participant 80

Participants described a variety of concerns that could apply regarding wet nursing. For example, they described how caregivers may worry about wet nurses outside their relatives, or the husbands/partners of wet nurses could sometimes be apprehensive about their children having enough milk or complain about wives leaving to feed others' infants. Family members could also worry that the nursed child becomes attached to the wet nurse. Misconceptions were among concerns such as a wet nurse may transfer bad traits, malnourished mothers cannot breastfeed or wet nurse, or concerns about the wet nurse having improper milk: “*There are myths and one myth like ‘Oh if the wet nurse is a bad person, she will transmit her bad personality!.”'* Participant 97

Other than their concerns about infection transmission, health professionals were usually thought to share the community's views, so whether they would support wet nursing was at least partly governed by the attitudes in their community. “*Health professionals … have attitudes that reflect the rest of the community. So, if breastfeeding and the sharing of bodily fluids is, umm, thought to be dangerous or revolting or, you know, sexualized or inappropriate in the community, then it's likely that health professionals will share those views.”* Participant 51

On a higher level, governments' willingness to facilitate NGO efforts was another factor that participants said needed to be considered. Some authority members may not understand how breastfeeding works, so they may not consider the importance of allocating assistance to breastfeeding mothers, like providing them with private spaces.

### Counseling and education approaches–special considerations

Participants drew attention to the point that providing breastfeeding counseling and information during difficult situations such as natural disasters must take into account the case of survivors who may be in a vulnerable psychological state after going through terrible conditions and perhaps losing loved ones. Therefore, the participants believed that breastfeeding information should be provided in such a way as to make it as easy as possible for mothers to understand. This might include using pictures to illustrate and translate the materials into the language of the survivors: “*… the mind of the survivors of the typhoons, half concentration half away… [We] make an announcement in a banner, and then in that banner, we put photos. And in that photo, we put captions in the language, in the [survivors'] dialect.”* Participant 97

In the situation described, all breastfeeding mothers were gathered in one tent, and in some cases, close relatives were integrated into the process of providing breastfeeding information, especially the mother-in-law, who has a special status in some societies. The participant found that this strategy not only facilitated communication but also gave a private space for breastfeeding mothers to breastfeed freely, away from the usual crowding in camp shelters. This positively affected wet nursing and made it easier to follow OG-IFE recommendations.

When facilitating wet nursing, it was noted that breastfeeding counselors need to provide special support to wet nurses because they will be breastfeeding infants who are often strangers to her, and if the infants are old enough, they can distinguish between the wet nurse and their mother. If the infant is dependent on artificial feeding, they may be uncomfortable with the unfamiliar breast nipple. These factors may complicate the breastfeeding process, and experienced counselors should support the wet nursing mother in managing these situations: “*…we will cluster the babies who are below five months because babies above five months already have a sense of familiarity; a new face, a new place. They sense of smell, the sense of voice. That's why we always say to the wet nurse, ‘Quiet, don't talk because the baby will know the voice.”'* Participant 97

An important point raised by some participants is that wet nursing mothers may have emotional responses when wet nursing. They described how some wet nurses developed an emotional bond with the infant, and in a unique case, a woman felt some aversion when she was wet nursing a child whose mother was too sick to breastfeed her own child. However, this wet nurse was motivated by her desire to help the sick mother. The wet nursing counselor should consider this emotional interaction by giving appropriate psychological support if the wet nurse is affected by these factors: “*For me, what I can say is that separation was very emotionally difficult because I became very attached to that baby.”* Participant 10

A participant noticed that there can be different medical recommendations offered to women, which may be incorrect (like hepatitis C infection is a contraindication to breastfeeding) or difficult or not culturally acceptable (such as using a cup or bottle for feeding expressed breastmilk). This can result in confusion and women not always following recommendations.

Participants felt it can assist wet nursing facilitation if the public is educated about breastfeeding importance with ongoing practical breastfeeding education and in a form that reaches people. One experienced breastfeeding advocate advised using social media in the education process: “*[The public] don't read anymore. They are a visual reader. They would look more at the television. Now, they have cell phones, at least in a family. They have one cell phone. They are now on the cell phone. They are now into TikTok. So, the conversation is TikTok, way entertaining.”* Participant 97

### Planning for implementation

As our participants dealt closely with wet nursing practice in general and humanitarian emergencies, they gave detailed insights into why and how wet nursing should be appropriately planned and implemented.

### Thorough planning matters

A common view among interviewees was that emergency preparedness is critical for the successful implementation of wet nursing as an infant feeding intervention via clear practical guidelines: “*Therefore, now that there is no calamity yet, although there is a crisis every day that needs breastfeeding help, we have to have breastfeeding preparedness and training.”* Participant 97

“*I think as a preparedness measure, this would need to be set up in countries before an emergency hits… So, when an emergency hits, we know how to do this, we know what the tests are we need to do or whatever.”* Participant 31

Several participants reported that there was coordination with different levels of local and national authorities including the military, ministry of health, local non-government organizations (NGOs), and community and religious leaders. In some cases, there were collaborations among NGOs and breastfeeding advocates from different countries where they helped translate available training materials into the local language. As part of planning, some participants suggested that those collaborations can help in mitigating some barriers by raising and educating the public. NGOs had a crucial role in improving breastfeeding rates in general while involving religious leaders was seen as beneficial. For example, this could help address Muslims' concerns about milk kinship: “*I think if there's a [religious] scholar who can also talk more about this arrangement, how it works, how it used to be in our Prophet's era, it also will be helpful so that people won't be afraid about the idea of wet nursing and the kids won't get married to each other or something like that.”* Participant 63

As with any intervention, implementing good breastfeeding practices, including wet nursing, requires resources such as breastfeeding counseling trainers for whom financial resourcing would be required. One participant mentioned the economic evaluation of the intervention with a comparison with the cost of infant formula during and after the emergency: “*If you do identification of resources, of course, for every activity you have a corresponding resource. But if you look into the local resources, not much, not expensive, but of course there will be incurred expenses. Who will be giving training? Of course, it's a work value, but when we come to the explanation of economics, like how much would you spend for the purchase of milk in a household, in a family, then they realize.”* Participant 97

### Infant formula control

It was noticed that successful wet nursing experiences in both general and humanitarian emergencies were associated with the absence of infant formula and strict rules applied to its use: “*Milk was not allowed in the camps because any powder at the time […] Water was not clean in those camps. […] So, the woman was illiterate to be able to sort of like, mix the powder. They didn't know how much; they didn't know how to read what was on the can. So, basically, it was not allowed. [We] had a very strict no-milk policy at the time in the camps.”* Participant 31

### Practical considerations

Some participants discussed resources and logistics. One commonly mentioned barrier was the paucity of trained emergency workers who had adequate knowledge of good breastfeeding practices, including wet nursing: “*But if you are thinking in most emergencies, there aren't skilled people around, they aren't policy experts.”* Participant 80

Some emergency response workers found that implementing good practices was overwhelming in such chaotic situations. As a result, human resources would be another factor to consider while planning for implementation: “*We did some internal capacity building for all the personnel and other organizations who were working in that reception center because we wanted to make sure, first of all, that nobody is doing this untargeted distribution of breast milk substitutes.”* Participant 43

Some detailed considerations were illustrated, like where the infant would be breastfed, distances between wet nurses' and caregivers' residents, and night feedings. Some participants raised the point of providing more than one wet nurse for each child to distribute the efforts: “*We had to look for a second wet nurse to do the second part-time because for example, let's say she has to breastfeed five times during the day. So, the first wet nurse would come three times in the morning and then another wet nurse would come in the afternoon… we tried to find ways to adjust so that the women don't feel they're doing it all by themselves, like coming many, many times.”* Participant 31

Another consideration was providing fresh food to disaster survivors in general and breastfeeding mothers in particular. Providing extra food to wet nurses recognizes that their time is valuable. However, participants emphasized that this must be applied with caution to avoid any kind of coercion of women by family members to be wet nurses to get the incentives.

### Identified facilitators and barriers

From these themes, different factors (facilitators and barriers) that affect wet nursing in emergencies were inferred and summarized in Table 2.

**Table 2:** Facilitators and barriers of wet nursing in emergencies with the related theme.

**Facilitators of wet nursing**	**Related theme**
• Understanding the importance of breastfeeding.	Breastfeeding is valued
• The practice is common in the community. • Community members are familiar with the practice. • Community cohesion and the desire to help other mothers (women-to-women support). • Availability of lactating relative or close friend. • Experienced mothers with older children are more willing to wet nurse. • The presence of milk sharing and milk banks. • Coordination with local authorities.	All stakeholders' acceptance Positive attitudes
•Availability of lactation counseling services. • Availability of experienced counselors who can mediate wet nursing and support wet nurses.	Counseling approach
• Providing private breastfeeding spaces. • Strictly targeted formula distribution. • All needed resources are available.	Planning for implementation
**Barriers to wet nursing**	**Related theme**
• Formula preferences and convenience. • Ignorance of formula risks. • Conflicting medical advice. • Misconceptions about breastfeeding.	Infant formula is normalized
• Fear of infection transmission especially among healthcare providers.	Infection transmission risk
• Western views of breastfeeding as an intimate activity. • Islamic rules of milk kinship. • Concerns of nursed child's family such as where the child will be nursed and the wet nurse's health. • Complains of the wet nurse's partner (e.g. about night feeding) and their concern about the wet nurse's child receiving enough care.	All stakeholders' acceptance Negative Attitudes
•Limited knowledge of IYCF-E among emergency workers.	Planning for implementation

## Discussion

### Main discussion

In this study, we aimed to understand the facilitators and barriers of wet nursing practice especially in emergencies, and find practical solutions to potential challenges by examining real-world experiences of women who were directly involved in wet nursing. This study reveals that factors that generally affect maternal breastfeeding in emergencies also influence wet nursing. Some of these factors have been identified in previous research on IYCF-E. Understanding the breastfeeding importance and infant formula risks, especially during emergencies is a crucial facilitator not only of maternal breastfeeding but also of wet nursing (16–18). Untargeted formula distribution is a common barrier to good breastfeeding practices in emergencies and strict policies for handling formulas in the refugee camps were found to help facilitate wet nursing (19). Introducing breastfeeding-friendly spaces is a common IYCF-E intervention and most wet nursing experiences reported in this study took place in breastfeeding-friendly spaces (16, 18). It is worth noting that our participants mostly reflected on successful experiences of practicing and implementing wet nursing as part of IYCF-E.

Our research identified three unique factors affecting wet nursing. They are the risk of infection transmission, cultural acceptability, and the need for specific counseling approaches. These factors are similar to previous literature on wet nursing, and the risk of disease transmission and cultural acceptability have been the most discussed factors throughout the history of wet nursing until the present (20). However, as medicine has progressed and the types of infection that can be transmitted through breastfeeding have been further defined, the risk factors, cultural factors and motives of practicing wet nursing have changed (20).

This study's findings build on published literature on wet nursing in emergencies (1, 14). Azad et al. (1) and Burrell et al. (14) analyzed the wet nursing experience during the Rohingya refugee crisis in Bangladesh in 2017. They identified several barriers including milk kinship (Muslim community), poor compliance between the wet nurses' and the nursed children's families, logistical obstacles such as night feeds and caregivers' preferences for infant formula (1, 14). Counseling by community nutrition workers and religious leaders was the key mitigation strategy for these challenges (1, 14). Including diverse cultures, our study results expand on these factors with attitudes of Western communities, healthcare professionals and health authorities, fear of infection transmission and the need for trained emergency workers. These results emphasize the crucial role of good knowledge of the importance of breastfeeding and the risks of infant formula among all stakeholders involved in wet nursing support during emergencies. Our findings here, also emphasize the importance of close monitor and support for wet nurses and the families of infants. For example, in case that an infant is being wet nursed by more than one woman or where wet nurse and infant are separated by some physical distance, continuous counseling would ensure that infant health is protected.

## Implications

### Implications for practice

There are a number of implications for practice that can be drawn from the study findings, particularly from the data within the “Counseling and education approaches- special considerations” and the “Planning for implementation” themes. These implications can be grouped together under three broad categories: planning and policies, knowledge and monitoring and evaluation of interventions

- Planning and policies:

Coordination between different levels of policymakers is crucial to formulating standardized guidelines and unifying messages sent to the public (19). Multisectoral collaboration is needed to enable comprehensive approaches and information materials (6). This collaboration might include national and international NGOs, health ministries, epidemiologists, and reproductive health specialists.

Tailored programs should be well-designed and include aspects of planning, implementation and evaluation (29). Collecting information about community experiences, beliefs and practices is needed to enable identification of cultural norms, religious rules, misconceptions, and resources (17). Breastfeeding-friendly spaces should be adopted as a standard strategy in all national policies and guidelines (16, 18, 30). Plans should consider managing challenges such as night feeding by providing simple containers for storing expressed breast milk and distances between wet nurses and nursed children's residences by accommodating the families of breastfed infants in close quarters to facilitate communication and transportation (14, 31). HIV testing kits should be considered as a resource, especially in prevalent areas and healthcare providers, counselors and emergency workers should have training in HIV risk assessment (6).

- Knowledge:

Effective dissemination of IYCF-E guidance related to breastfeeding and wet nursing should be worldwide and translated into different languages to ensure reaching targeted groups such as healthcare professionals and emergency workers (32, 33).

Educational programs should be provided even before disaster strikes and should include information on the importance of breastfeeding and the risks of infant formula for the public, infection control during emergencies for healthcare professionals, and general knowledge of IYCF-E and OG-IFE for emergency workers (34). Further training may be needed during the emergency response for emergency responders and volunteers (34).

Training materials for volunteers and specific wet nursing counseling should be prepared as simple infographics and translated into different languages to be ready for immediate usage.

Breastfeeding counselors should be trained on specific wet nursing requirements such as mediation between families and how to train and support wet nurses and their children (32).

Concerns of milk kinship can be managed by involving local Islamic scholars and Imams in educating Muslim communities on how to record personal details for both the wet nurse and the nursed child and may suggest continuous communication between the families to keep the connection between milk siblings (1, 25).

- Monitoring and evaluation of interventions

While data collection during emergencies may be challenging, it is vital for evaluating short and long-term outcomes of wet nursing and standardized forms should be formulated as part of the planning stage (16, 33). Along with breastfeeding indicators, outcome indicators may also include infants' morbidity and mortality rates, children's nutritional status and the effectiveness of psychological support to mothers (17).

Cost-benefit evaluations of breastfeeding, including wet nursing, in different contexts are needed to assist in justifying the resources required to support breastfeeding and wet nursing, including trained human resources. A recently published cost analysis in two contexts found a substantial difference in costs between supporting exclusive maternal breastfeeding and breastmilk substituents usage in humanitarian emergencies (35). However, a cost-benefit analysis of wet nursing has not been considered.

### Implications for research

As previously described, literature on wet nursing in contemporary settings is scarce. It is noted that nationwide surveys like Demographic and Health Surveys (DHS) and UNICEF Multiple Indicator Cluster Survey (MICS) do not separate “breastfed from another woman” in breastfeeding indicators (33). Data on the prevalence of wet nursing practice in each country should be collected in these nationwide nutrition surveys. This would also improve recognition of the practice and may ultimately improve acceptance.

While HIV transmission has been thoroughly studied, further investigation is needed for the risk and outcomes of HTLV-1 and CMV in relation to wet nursing and cross-feeding in standard conditions as part of preparedness planning because such research projects may not be feasible in emergency settings (36, 37).

As this is a qualitative study, we did not aim to generalize our findings but to discover phenomena that may be associated with wet nursing practice. Quantitative surveys should examine the identified facilitators and barriers and determine the cultural differences between communities. The results of these surveys would be crucial to designing tailored educational programs that support good breastfeeding practices including wet nursing.

## Strengths and limitations

The strength of this study lies in our unique participants who shared their professional and personal experiences of wet nursing and gave their invaluable insights into lessons learned.

However, there are some limitations including the small sample size. Recruitment was a challenge as participants who could not communicate in English were not recruited because of translation challenges in online interviews. The general population may have a different perspective on wet nursing from our participants who were breastfeeding experts and reflected mostly on positive experiences. As mentioned, our findings cannot be generalized but can be tested in different countries. In addition, some participants reflected on past experiences, so results can be prone to recall bias. There is room for further qualitative research on this subject.

## Conclusion

Promoting wet nursing is an IYCF-E recommendation and is in line with supporting good breastfeeding practices in emergencies. Wet nursing can be facilitated when an experienced counselor mediates it, breastfeeding is valued, and the community is familiar with wet nursing practice. However, some barriers may specifically influence wet nursing, including the risk of infection transmission, negative attitudes toward wet nursing and the need for culturally sensitive counseling to mediate wet nursing. We provided some detailed recommendations to mitigate these barriers. This study's findings can guide further research on the practice and most importantly, guide practical actions for implementing wet nursing in different settings.

## Data Availability

The datasets presented in this article are not readily available because the dataset consists of interviews transcripts that cannot be shared for participants' confidentiality. Requests to access the datasets should be directed to Seema Mihrshahi, seema.mihrshahi@mq.edu.au.
