# Supplementary material for: Facilitators and barriers of wet nursing: a qualitative study with implications for emergencies
Source: Front Nutr. 2025 May 9;12:1456675. doi: 10.3389/fnut.2025.1456675 (PMC12098040; doi:10.3389/fnut.2025.1456675)
Supplement: Supplementary file 1 [file Data_Sheet_1.pdf]

Supplementary File 1

Interview guide

Wet nurses

After confirming that the participant fully understands the PICF and obtaining verbal consent from the participant,

If you are comfortable with this, I will start the interview questions:

Can you describe your experience with wet nursing?

What factors were most helpful for the wet nursing experience?

If necessary, prompt in terms of gender, cultural, religious facilitators, etc.

What factors were most hindering to the wet nursing experience?

If necessary, prompt in terms of gender, cultural, religious barriers, etc.

What do you think would have helped with these factors?

Breastfeeding Counsellors

After confirming that the participant fully understands the PICF and obtaining verbal consent from the participant,

If you are comfortable with this, I will start the interview questions:

Have you ever witnessed, or heard about wet nursing? On what occasion?

What factors are most helpful for the wet nursing practice?

OR what factors make wet nursing a viable/positive/desirable practice?

If necessary, prompt in terms of gender, cultural, religious, policy facilitators, etc.

What factors are most hindering the wet nursing practice?

OR what factors make wet nursing a less viable/less desirable practice?

If necessary, prompt in terms of gender, cultural, religious, policy barriers, etc.

What do you think would help with these factors?
